# Accelerated 3D multi-channel ${\text{B}}_{1}^{+}$ mapping at 7 T for the brain and heart

**DOI:** 10.1002/mrm.30201

**Published:** 2024-06-27

**Authors:** James L. Kent, Matthijs H. S. de Buck, Iulius Dragonu, Mark Chiew, Ladislav Valkovič, Aaron T. Hess

**Affiliations:** 1 Wellcome Centre for Integrative Neuroimaging, FMRIB, Nuffield Department of Clinical Neurosciences, University of Oxford, Oxford, UK; 2 Spinoza Centre for Neuroimaging, Amsterdam, The Netherlands; 3 Computational Cognitive Neuroscience and Neuroimaging, Netherlands Institute for Neuroscience, KNAW, Amsterdam, The Netherlands; 4 Department of Radiology and Nuclear Medicine, Amsterdam University Medical Centers, University of Amsterdam, Amsterdam, The Netherlands; 5 Research & Collaborations GB&I, Siemens Healthcare Ltd, Camberley, UK; 6 Department of Medical Biophysics, University of Toronto, Toronto, Ontario, Canada; 7 Physical Sciences, Sunnybrook Research Institute, Toronto, Ontario, Canada; 8 Oxford Centre for Clinical Magnetic Resonance Research (OCMR), University of Oxford, Oxford, UK; 9 Department of Imaging Methods, Institute of Measurement Science, Slovak Academy of Sciences, Bratislava, Slovakia

**Keywords:** B_1_^+^ mapping, B1TIAMO, parallel transmit, TxLR, ultrahigh field MRI

## Abstract

**Purpose:**

To acquire accurate volumetric multi-channel ${\text{B}}_{1}^{+}$ maps in under 14 s whole-brain or 23 heartbeats whole-heart for parallel transmit (pTx) applications at 7 T.

**Theory and Methods:**

We evaluate the combination of three recently proposed techniques. The acquisition of multi-channel transmit array ${\text{B}}_{1}^{+}$ maps is accelerated using transmit low rank (TxLR) with absolute ${\text{B}}_{1}^{+}$ mapping (Sandwich) acquired in a ${\text{B}}_{1}^{+}$ time-interleaved acquisition of modes (B1TIAMO) fashion. Simulations using synthetic body images derived from Sim4Life were used to test the achievable acceleration for small scan matrices of 24 × 24. Next, we evaluated the method by retrospectively undersampling a fully sampled ${\text{B}}_{1}^{+}$ library of nine subjects in the brain. Finally, Cartesian undersampled phantom and in vivo images were acquired in both the brain of three subjects (8Tx/32 receive [Rx]) and the heart of another three subjects (8Tx/8Rx) at 7 T.

**Results:**

Simulation and in vivo results show that volumetric multi-channel ${\text{B}}_{1}^{+}$ maps can be acquired using acceleration factors of 4 in the body, reducing the acquisition time to within 23 heartbeats, which was previously not possible. In silico heart simulations demonstrated a RMS error to the fully sampled native resolution ground truth of 4.2° when combined in first-order circularly polarized mode (mean flip angle 66°) at an acceleration factor of 4. The 14 s 3D ${\text{B}}_{1}^{+}$ maps acquired in the brain have a RMS error of 1.9° to the fully sampled (mean flip angle 86°).

**Conclusion:**

The proposed method is demonstrated as a fast pTx calibration technique in the brain and a promising method for pTx calibration in the body.

## 1 Introduction

Ultrahigh field MRI promises superior SNR over conventional clinical MR systems.^1^ However, at 7 T, ${\text{B}}_{1}^{+}$ inhomogeneity is large across the brain and severe across the heart, limiting the utility of ultrahigh-field quantitative cardiac imaging. Parallel transmit (pTx)^2,3^ can solve this problem^4–6^ but requires multi-channel ${\text{B}}_{1}^{+}$ maps for optimal RF pulse design and RF shimming. Multi-channel ${\text{B}}_{1}^{+}$ maps in the heart are difficult to acquire in a clinically reasonable timeframe and are confounded by issues of cardiorespiratory motion, blood flow, and large B_0_ off-resonances. Because ${\text{B}}_{1}^{+}$ maps are generally only required for calibration before scanning, their acquisition should be as fast as possible and ideally well under a minute. Whereas accurate maps are essential, high-resolution maps are not usually necessary because the wavelength associated with the Larmor frequency is approximately 11 cm at 7 T. Free-breathing approaches have been demonstrated,^7^ but these require acquisition and reconstruction times of several minutes. The efficacy of measuring ${\text{B}}_{1}^{+}$ maps during diastole and applying the calculated dynamic RF pulses throughout the entire cardiac cycle has been shown.^4^

Relative ${\text{B}}_{1}^{+}$ maps are generally fast to acquire,^8^ taking less time than absolute ${\text{B}}_{1}^{+}$ mapping. Recent approaches have shown the ability to estimate relative maps from localizers,^9^ but an absolute measure of ${\text{B}}_{1}^{+}$ is required to achieve a specific flip angle. Absolute ${\text{B}}_{1}^{+}$ mapping using 3DREAM^10,11^ works well in the head and can acquire high-resolution eight-channel ${\text{B}}_{1}^{+}$ maps in approximately 1 min. However, DREAM-^12,13^ based ${\text{B}}_{1}^{+}$ mapping is undesirable for use in the heart due to sensitivity to flow.^14,15^ Presaturation TurboFLASH^16^ is ideal for ${\text{B}}_{1}^{+}$ mapping in the heart due to its inherent insensitivity to flow but has a strong T_1_ dependency.^17^ Additionally, presaturation TurboFLASH suffers from long acquisition times due to the long time delay of 5T_1_ required between the reference and prepared images. We have previously shown that using short imaging trains and “sandwiching” the reference and prepared images together overcomes both of these problems.^18^ The use of a nonadiabatic hyperbolic secant (HS8) pulse for presaturation also achieves better B_0_ insensitivity over a conventional sinc or rectangular preparation pulse, thereby requiring no additional B_0_ correction.^19^ These simple pulse sequence modifications allowed for faster acquisition of the absolute ${\text{B}}_{1}^{+}$ maps and, as a result, we demonstrated single breath-hold whole-heart 3D ${\text{B}}_{1}^{+}$ mapping for a single shim mode.^18^

Recently, an acquisition strategy known as ${\text{B}}_{1}^{+}$ time-interleaved acquisition of modes (B1TIAMO)^20,21^ was proposed, which acquires fast relative multi-channel maps and two absolute ${\text{B}}_{1}^{+}$ maps with complementary shim modes. The two absolute shim mode maps are used to help overcome the limited dynamic range by ensuring sufficient ${\text{B}}_{1}^{+}$ in at least one of the shim modes and to update the multi-channel relative maps to absolute units. B1TIAMO has been shown to obtain accurate 2D multi-channel maps for body imaging in 16 s for a 32-transmit (Tx) system.^20^ Furthermore, Hess et al. recently proposed an algorithm for reconstructing undersampled calibrationless Tx field maps (transmit low rank [TxLR])^22^ that could allow for acceleration factors of up to 8 in the body, matching the number of transmit channels. Hess et al. also showed that the minimum matrix size for capturing the dominant transmit modes in the heart was 24 × 24. Hence, TxLR enables eight-channel transmit 3D relative maps with 24 × 24 × 8 lines of k-space data to be acquired in only 2 s or five heartbeats (TR = 3.5 ms, cardiac window = 400 ms).

Therefore, in this work, we evaluate TxLR in combination with a B1TIAMO-style acquisition and fast absolute maps using the Sandwich method^18^ to acquire accelerated 3D multi-channel absolute ${\text{B}}_{1}^{+}$ maps in 14 s in the brain or 23 heartbeats in the body^23^. We evaluate this approach using in silico simulations; retrospective undersampling of 3D in vivo whole-head data from a library of nine subjects; and finally, prospectively undersampled data acquired in phantoms, the brain of three subjects, and the heart of another three subjects. This study is investigating a method to accelerate maps and is not proposing a new ${\text{B}}_{1}^{+}$ mapping method. Hence, to avoid repetition of previous work, the evaluation of the proposed method is largely focused on comparing to fully sampled data.

## 2 Methods

### 2.1 Acquisition scheme

**Figure 1:**
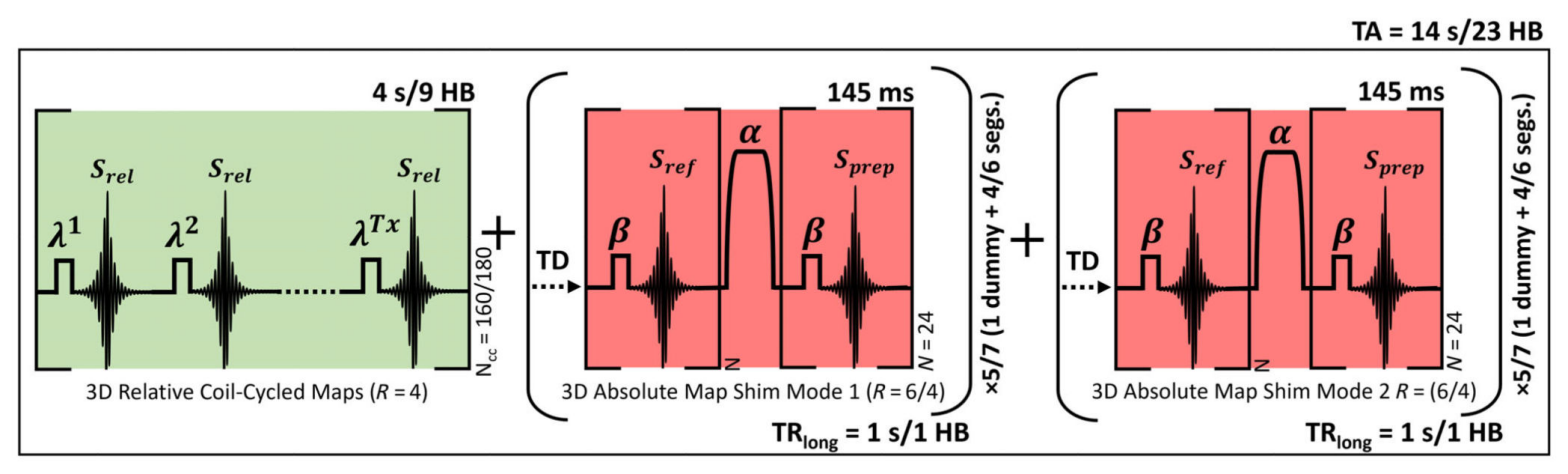
Schematic sequence diagram for acquiring fast multi-channel ${\text{B}}_{1}^{+}$ maps in the brain/heart. Firstly, multi-channel relative maps (green) are acquired for a number of coil-cycles (*N*_cc_), including 16 dummy coil-cycles. This is followed by absolute maps (red) using Sandwich for complementary shim modes: first- and second-order circularly polarized (CP^+^, CP^2+^) modes with a TurboFactor of *N*. The absolute maps excitation (*β*) and 5 ms nonadiabatic hyperbolic secant (order 8) preparation pulse (*α*) have matched transmit mode. The sequence can be adapted for cardiac ${\text{B}}_{1}^{+}$ mapping by segmenting the relative maps into 9 HBs and acquiring the absolute maps with TR_long_ = 1 HB. Brackets indicate repeated sequence blocks. HB, heartbeat; *R*, acceleration factor; TA, total acquisition; TD, delay time.

The proposed acquisition scheme is shown in Figure 1. In a B1TIAMO^20^ fashion, we acquire 3D coil-cycled multi-channel relative maps using a spoiled low flip angle gradient echo (GRE) sequence followed by two absolute maps with complementary orthogonal shim modes (first- and second-order circularly polarized, CP^+^ and CP^2+^, respectively) using the modified Sandwich presaturation TurboFLASH sequence.^18^

### 2.2 Numerical heart simulations

Simulations were performed using retrospectively undersampled in silico data to establish the achievable acceleration. The in silico body ${\text{B}}_{1}^{+/-}$ fields were simulated in Sim4Life 3.4 (ZMT, Zurich, Switzerland) using an eight-channel transmit/receive (Tx/Rx) dipole array^24^ centered over the heart of Duke (Virtual Population, ITIS Foundation, Zurich, Switzerland) at 7 T.^22^ A synthetic proton density image was generated by setting the proton density equal to the tissue density, with tissue densities greater than 1200 kg/m^3^ (bone) or <400 kg/m^3^ (lung) set to 80 to make them resemble an MR image. The FOV was 278 × 356 mm^2^ (anterior-to-posterior/left-to-right). Data were re-sampled onto a uniform grid of 2 × 2 mm^2^. The relative (*N*_Tx_ = 8) and reference (*N*_Tx_ = 2) images were formed from a multiplication of these proton-density weighted images and either individual transmit ${\text{B}}_{1}^{+/-}$ or CP^+^ and CP^2+^. The prepared (*N*_Tx_ = 2) images are formed from the multiplication of the reference images with the cosine of the ${\text{B}}_{1}^{+/-}$ for CP^+^ and CP^2+^. Rx channel images were then formed from the multiplication of the relative, reference, and prepared images by the synthetic coil-sensitivities. The synthetic (Rx coil-combined) images are shown in Figure S1.

The synthetic body images were inverse fast Fourier transformed and k-space corrupted with channel-independent complex Gaussian noise (peak SNR 60 dB). The k-space data was cropped to a matrix size of 24 × 24; which was previously found to be the minimum scan matrix for capturing the dominant transmit modes in the heart.^22^ This was revalidated by comparing the relative error between k-space cropped (24 × 24, Hann filtered and zero-padded back to 139 × 178) and native resolution (139 × 178) ground truth relative and absolute maps.

Homogenous Poisson-disc undersampling masks were generated using SPIRiT Toolbox v0.3 for effective undersampling factors between 1 and 10. To evaluate the impact of the absence of a calibration region, masks were also generated both with and without a central 4 × 4 calibration region. A different undersampling mask was used for each transmit mode, with the reference and prepared images of the absolute maps using the same undersampling mask.

The relative (*N*_Tx_ = 8), reference (*N*_Tx_ = 2), and prepared (*N*_Tx_ = 2) undersampled k-spaces were concatenated to form a 24 × 24 × 8 × 12 matrix (*N*_kx_ × *N*_ky_ × *N*_Rx_ × *N*_Tx_) and jointly reconstructed using the TxLR algorithm (5 × 5 kernel, 50 iterations, and rank threshold of 50). The reconstructed k-space data were Hann filtered, zero-padded up to 139 × 178, and fast Fourier transformed back to image space. The Rx channel images were combined using Rx sensitivity maps estimated from the relative map reconstructed k-space using ESPIRiT (5 × 5 kernel, 0.02 eigen threshold).^25^ ESPIRiT was also used to calculate the transmit sensitivities. The flip angle for the absolute maps was found from the arccosine of the reference and prepared image ratio.

The individual multi-channel maps were then calculated based on the method proposed by Brunheim et al. with a total scaling exponent *m* = 4, as defined in the original B1TIAMO paper,^20^ and weighted based on the reference image intensity. Maps were masked from a simple binary value-thresholding based on the fully sampled relative images. Simulations were repeated 50 times with each repetition using unique noise and undersampling masks. To assess their accuracy within the heart, the difference in the reconstructed multi-channel maps to the fully sampled native resolution (noiseless) ground truth is shown as well as the RMS error (RMSE) for maps combined into CP^+^ mode.

### 2.3 Parallel transmit MRI system

A 7 T Magnetom (VB17, Siemens Healthcare, Erlangen, Germany) equipped with pTx was used for phantom and in vivo imaging. Brain images were acquired using an 8Tx/32Rx head coil (Nova Medical, Wilmington, USA) using fixed per-channel power limits as provided by the coil manufacturer of 1.5 W per channel. To image the heart an 8Tx/8Rx dipole array (MR Coils BV, Zaltbommel, The Netherlands) was used with internally approved power limits based on simulation and measurement for a worst-case per channel limit of 3.98 W. Cardiac triggering was performed using a pulse oximeter (Siemens Healthcare) with a trigger delay of 100 ms.

### 2.4 Retrospective undersampling of brain data

Using a similar processing pipeline as outlined above, fully sampled 3D relative multi-channel maps and absolute ${\text{B}}_{1}^{+}$ maps for CP^+^ and CP^2+^ in the brain were retrospectively undersampled from a database of nine subjects (23–56 years old; seven male/two female) acquired by de Buck et al.^26,27^ This database was acquired using a similar sequence as outlined in the following sections but with the following sequence parameters. Both relative and absolute sequences used a 300 × 225 × 225 mm^3^ FOV with a reference voltage of 50 V (per channel). The relative maps were acquired using a GRE sequence with TE_GRE_ = 1.02 ms, TR_GRE_ = 2.90 ms, bandwidth = 500 Hz/px, RF spoiling. A scan matrix of 40 × 36 × 36 and, nonselective rectangular RF excitation with a nominal flip angle of 7°. The scan time required for this relative sequence was 30 s. Absolute maps were acquired for two shim modes, which were CP^+^ and CP^2+^. TurboFLASH TE_FLASH_ = 1.78 ms, TurboFLASH TR_FLASH_ = 3.92 ms, TR_long_ = 1 s, bandwidth = 489 Hz/px, RF spoiling. A scan matrix of 48 × 27 × 36 was used with nonselective rectangular RF excitation (*β*). A 500 μs rectangular RF pulse was used for presaturation (*α*) (whereas a HS8 pulse is used in the current work), with an *α*:*β* ratio of 10:1, using nominal α/β flip angles 90°/9°. The scan time required for each shim mode map was 36 s for a combined scan time of 1 min 42 s.

### 2.5 Sequence implementation

Separate sequences were used to acquire the relative and absolute portions of the proposed method. 3D accelerated relative maps were acquired using a GRE sequence, coil-cycled to minimize the influence of magnetization history, with TE_GRE_ = 1.31 ms, TR_GRE_ = 3.14 ms, bandwidth = 490 Hz/px, RF spoiling, and an acceleration factor of *R* = 4. A scan matrix of 24 × 24 × 24 was used, nonselective rectangular RF excitation with a flip angle (*λ*) of 9°. The acquisition time was 4 s (*N*_cc_ = 160), of which the first 400 ms were 16 dummy coil-cycles. Undersampling masks were as described previously (section 2.2) and ordered as in Jaeschke et al.^28^ to minimize the eddy currents from a rapid change in the phase encoding. For cardiac mapping during diastole, the relative map k-space was segmented into nine heartbeats of 16 coil-cycles (400 ms). During the trigger delay, four dummy coil-cycles (100 ms) were performed (*N*_cc_ = 180).

3D accelerated absolute maps were acquired for CP^+^ and CP^2+^ using the Sandwich sequence^18^ with TurboFLASH TE_FLASH_ = 1.26 ms, TurboFLASH TR_FLASH_ = 3 ms, bandwidth = 490 Hz/px, and RF spoiling. A scan matrix of 24 × 24 × 24 was used with nonselective rectangular RF excitation (*β*). A 5 ms nonadiabatic HS8^19,29^ RF pulse was used for presaturation (*α*) using nominal *α*/*β* flip angles 90°/5°. Different acceleration factors were used in the brain/heart for the acquisition of the absolute maps, which were *R* = 6/4, respectively. Each absolute map was segmented into 4/6 (*R* = 6/4), one segment per TR_long_ = 1 s, to maintain a readout train of 24 phase encoding lines (TurboFactor = 24, duration of 145 ms) per segment. The acquisition time was 5 s (*R* = 6) or seven heartbeats (*R* = 4) per map, which includes 1 dummy TR_long_. Hence, a total of 10 s or 14 heartbeats for both mode maps. Undersampling masks were as described previously (section 2.2) (see Figure S2). A square-spiral acquisition order was used, center-in for the reference images and center-out for the prepared images to reduce the impact of T_1_ on the image trains. Although separate sequences were used, the total time to acquire both the relative and absolute maps is 14 s ungated or 23 heartbeats gated. Table 1 summarizes the protocols used in phantom and in vivo.

### 2.6 Phantom experiments

3D multi-transmit ${\text{B}}_{1}^{+}$ maps were acquired in two coil/phantom combinations. The first was the 8Tx/32Rx head coil using a 16.5 cm diameter spherical MRS “Braino” water phantom (General Electric Medical Systems, Milwaukee, USA) (reference voltage 60 V), and the second was the 8Tx/8Rx chest coil using a large custom 27 cm diameter 73 mM NaCl drum phantom (reference voltage 200 V). The Braino phantom was acquired with a TR_long_ = 1 s using a 250 × 250 × 250 mm^3^ FOV, giving 10.4 × 10.4 × 10.4 mm^3^ resolution. The drum phantom was acquired gated with a simulated R-wave peak-to-peak interval of 700 ms. The FOV used for imaging the drum phantom was increased to 300 × 300 × 300 mm^3^, giving 12.5 × 12.5 × 12.5 mm^3^ resolution.

The offline reconstruction time was 5 min for the head coil and 1 min for the chest coil using 20 TxLR iterations. For reference, a 3D fully sampled B1TIAMO map was acquired with a matched scan matrix and FOV using fully sampled relative maps (15 s or 36 simulated heart-beats) and gated Sandwich absolute CP^+^ and CP^2+^ maps (50 s or 50 simulated heartbeats for both modes).

### 2.7 In vivo experiments

All subjects were scanned under an institutionally approved technical development standard operating procedure (FMRIB_004_V4). 3D multi-transmit maps were acquired in the brain of three volunteers at a reference voltage of 60 V as described above for the head coil phantom at acceleration factors of R = 4/6. The FOV for relative and absolute Sandwich maps was 250 × 250 × 250 mm^3^, giving 10.4 × 10.4 × 10.4 mm^3^ acquired resolution. The maps were reconstructed to 64 × 64 × 64 for plotting. A 3D fully sampled B1TIAMO dataset was acquired for reference in 65 s. We also tested the application of three coil-compression algorithms from the SPIRiT Toolbox v0.3 before TxLR reconstruction for a range of virtual coils 2–32.

3D cardiac multi-transmit maps were acquired in three healthy volunteers at a reference voltage of 200 V. The FOV for relative and absolute Sandwich maps was 380 × 380 × 285 mm^3^, giving 15.8 × 15.8 × 11.9 mm^3^ resolution. The maps were reconstructed to 64 × 64 × 48 for plotting. The coil-cycled relative maps and absolute maps were acquired in two separate breath-holds due to the sequence preparation time, both at end-expiration to reduce respiratory motion artifacts. However, in total 23 heartbeats (9 segments relative maps + 2 Sandwich dummys + 6 segments Sandwich CP^+^ + 6 segments Sandwich CP^2+^) were required for data acquisition. A fully sampled set of 3D B1TIAMO maps would have required 86 heartbeats.

### 2.8 Image data reconstruction and combination

Reconstruction was performed offline in MatLab (R2021a, MathWorks, Natick, MA, USA) using the parallel computing toolbox (14 workers) on an Intel Xeon 28 × 2.40 GHz 128Gb RAM computer running Rocky Linux 8.5. Relative, reference, and prepared k-space data were concatenated to form a 24 × 24 × 24 × (8/32) × 12 (*N*_kx_ × *N*_ky_ × *N*_kz_ × *N*_Rx_ × *N*_Tx_) matrix and jointly reconstructed using TxLR slice-by-slice on hybrid x-k_y_-k_z_ data. The k-space data were Tukey filtered (cosine fraction = 0.7), zero-padded to 32 × 32 × 32 (unless mentioned otherwise), and fast Fourier transformed to full image space. Using ESPIRiT, Rx channel images were combined using sensitivity maps estimated from relative map k-space. ESPIRiT was also used to calculate the transmit sensitivities.

The flip angle for the absolute maps was calculated from the ratio of the reference and prepared images using a lookup table generated from Bloch and extended phase graph simulations,^30^ which includes a correction for the nonlinearity of the HS8 pulse employed for saturation. The individual multi-channel maps were then calculated based on the method proposed in the B1TIAMO paper^20^ with a total scaling exponent *m* = 3, as defined in the original B1TIAMO paper, and weighted based on the reference image intensity. Maps were masked from a simple binary value-thresholding based on the fully sampled relative images. Voxels in the absolute maps outside of the lookup table were masked out and any holes were linearly interpolated. Voxels that had a maximum shim efficiency below 30%, as calculated from the relative maps, were additionally masked out due to low signal producing erroneous measurements of absolute ${\text{B}}_{1}^{+}$.

## 3 Results

### 3.1 Numerical heart simulation results

**Figure 2:**
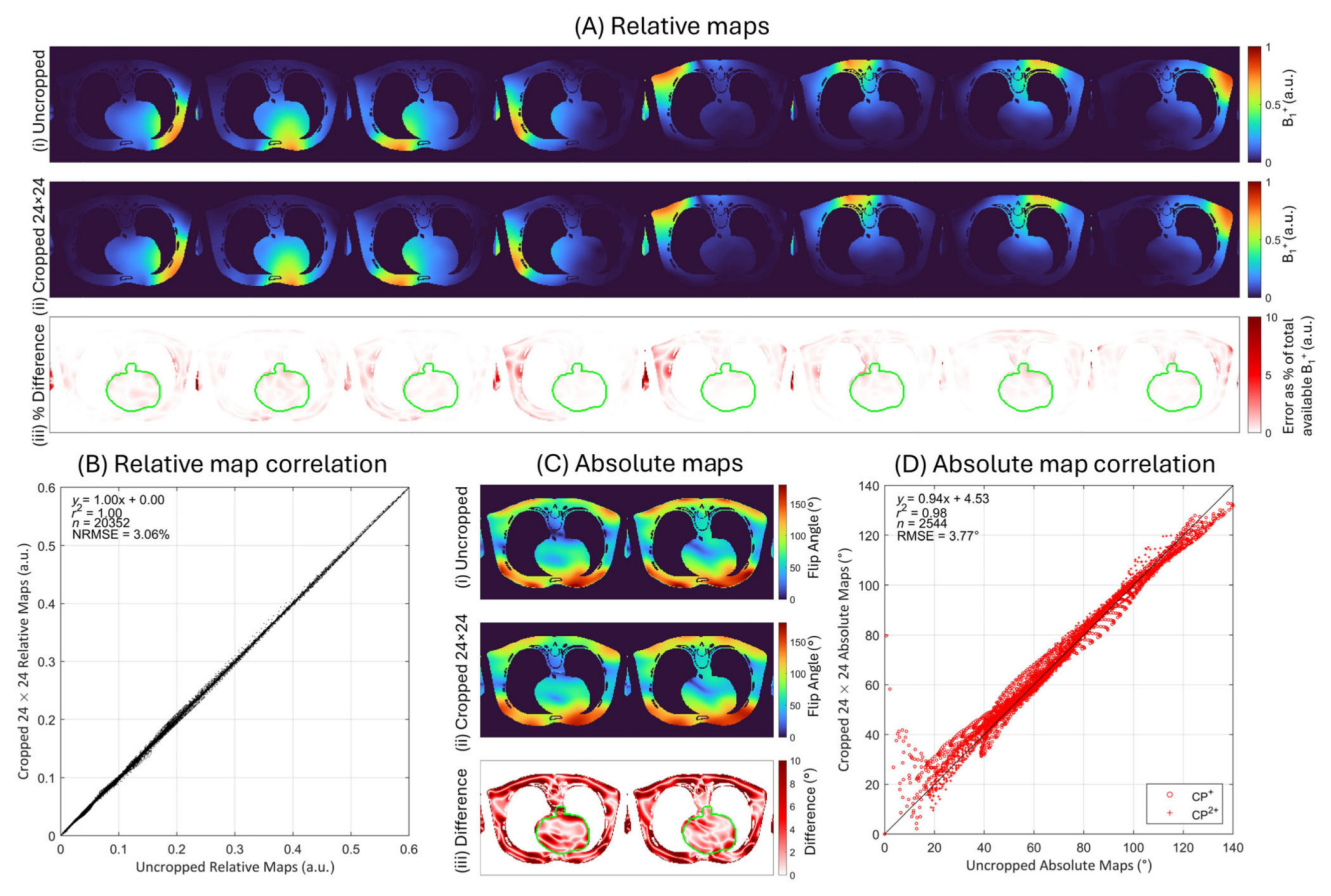
Comparison between the magnitude of the native resolution (139 × 178) and k-space cropped (24 × 24) ground truth for relative and absolute maps (CP^+^ and CP^2+^) of synthetic body data. The mean error between the cropped and native resolution relative maps as a percentage of the total ${\text{B}}_{1}^{+}$ available is 3.1% within the ROI (green line). The mean error between the cropped and native resolution absolute maps is 3.8° (mean flip angle in heart of 67°) for the absolute maps within the ROI (green line). CP, circularly polarized; ROI, region of interest.

Figure 2 shows the impact on relative and absolute maps of cropping k-space down to a central 24 × 24 region compared to the native resolution (139 × 178) ground truth. The normalized RMSE within the heart was 3.1% for relative maps and the RMSE 3.8° (mean flip angle 67°) for absolute maps with the largest errors close to regions of ${\text{B}}_{1}^{+}$ constructive/destructive interference. This indicates we can capture the dominant transmit modes in the heart using small scan matrices.

**Figure 3:**
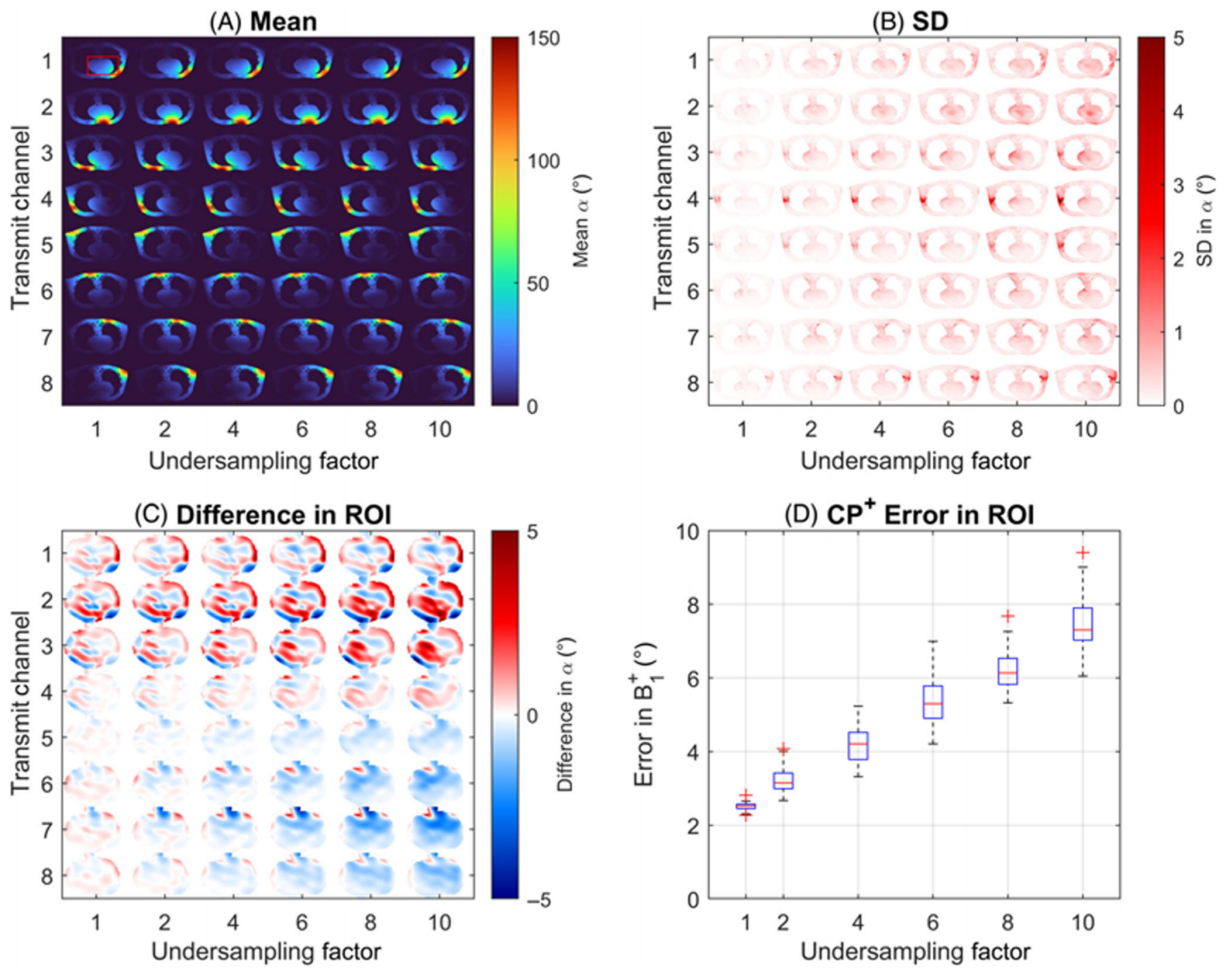
Simulation results from synthetic body images showing the mean (A) and SD (B) for multi-channel parallel transmit ${\text{B}}_{1}^{+}$ maps with undersampling factors of 1 to 10. The mean flip angle in the heart in CP^+^ mode was 66° (min/max = 6°/137°, CV = 36%). A total of 50 TxLR iterations and 50 repeats with different undersampling masks were performed. Subfigure (C) shows the mean absolute error across these repeats to the fully sampled native resolution (139 × 178) k-space in the ROI (red box in the top left of subfigure (A)). (D) RMSE calculated on the complex values for multi-channel maps combined in CP^+^ mode for the 50 repeats. CV, coefficient of variation; RMSE; RMS error; TxLR, transmit low rank.

Simulation results from synthetic 8Tx/8Rx body images for undersampling factors are shown in Figure 3. The mean flip angle and SD over 50 repeats each with different undersampling masks are shown in Figure 3(A,B). The mean flip angle within the heart in CP^+^ mode was 66° (min/max = 6°/137°, coefficient of variation [CV] = 36%). In Figure 3(C), large undersampling factors of 10 show increased error in the measured ${\text{B}}_{1}^{+}$ within the heart when compared to the fully sampled native resolution maps. Figure 3(D) shows a complex RMSE to the fully sampled native resolution ground truth of 4.2° combined in CP^+^ mode within the heart at an acceleration factor of 4. The RMSE increases linearly with undersampling factor.

Fully sampling the central 4 × 4 region of k-space had a negligible impact on the error in the reconstructed maps at undersampling factors of 4 (Figure S3). However, including a fully sampled central 4 × 4 region did improve the error in the reconstructed maps at higher undersampling factors, lowering the RMSE from 7.8° to 6.1° at an acceleration factor of 8. A total of 20 TxLR iterations appears optimal for acceleration factors up to 4 in the body, where the RMSE of the combined CP^+^ map to the native resolution ground truth was 4.3° in the heart (5.0° whole-body). The fully sampled had an RMSE of 2.5° in the heart (3.1° whole-body) (Figure S4).

### 3.2 Retrospective undersampling of brain data results

**Figure 4:**
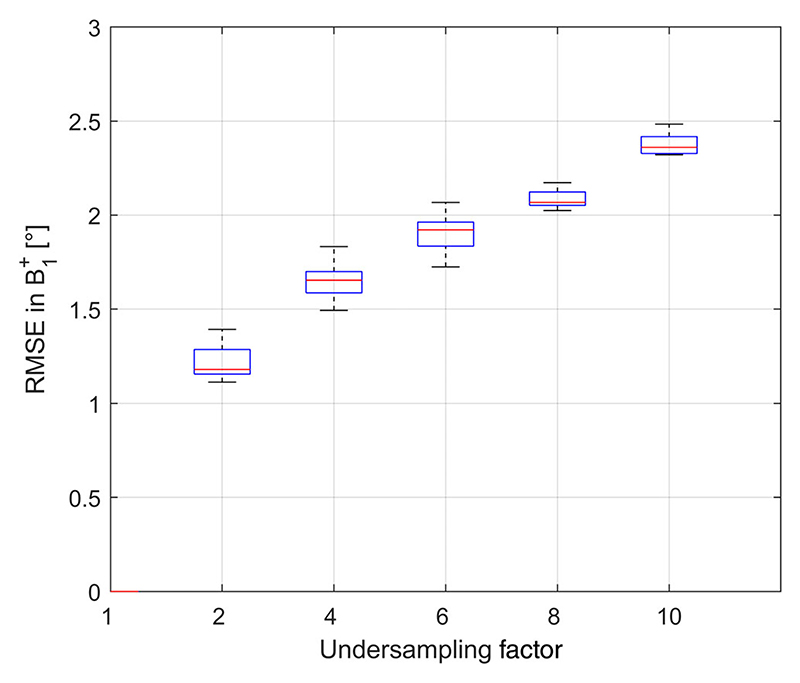
Boxplots showing the complex RMSE for channels combined into CP^+^ (mean flip angle in CP^+^ was 45°, min/max = 11°/88°, CV = 29%) for various retrospective undersampling factors. In vivo data using a ${\text{B}}_{1}^{+}$ library of 3D brain data from nine subjects.

The results of the retrospective undersampling of nine subjects using the head coil are shown in Figure 4. The mean flip angle in the brain in CP^+^ was 45° (min/-max = 11°/88°, CV = 29%), with the RMSE increasing with increased acceleration factors. Acceleration factors of 4, 6, and 8 gave an average RMSE for multi-channel maps combined into CP^+^ of 1.7°, 1.9°, and 2.1°, respectively.

### 3.3 Phantom results

Figure S5 shows ${\text{B}}_{1}^{+}$ maps obtained in the “Braino” phantom and the difference compared to the ground truth fully sampled maps. The mean flip angle across the entire phantom was 90° in CP^+^ (min/max = 34°/175°, CV = 21%), with an RMSE of 3.7° to the fully sampled.

Figure S6 similarly shows the synthetically gated ${\text{B}}_{1}^{+}$ maps obtained in the large drum phantom and the difference to the ground truth fully sampled maps. The mean flip angle across the entire drum phantom in CP^+^ was 60° (min/max = 0°/214°, CV = 45%), with an RMSE of 3.5°. A large number of phase ramps are visible in Figure S6 due to the high permittivity of the phantom. There are some artifacts that appear in the ${\text{B}}_{1}^{+}$ maps, which are explained by the choice of complementary shims. Figure S7 shows the shim efficiencies and confirms that these errors are pronounced where the maximum shim efficiency is below 30%, as indicated by the white arrows.

### 3.4 In vivo results

**Figure 5:**
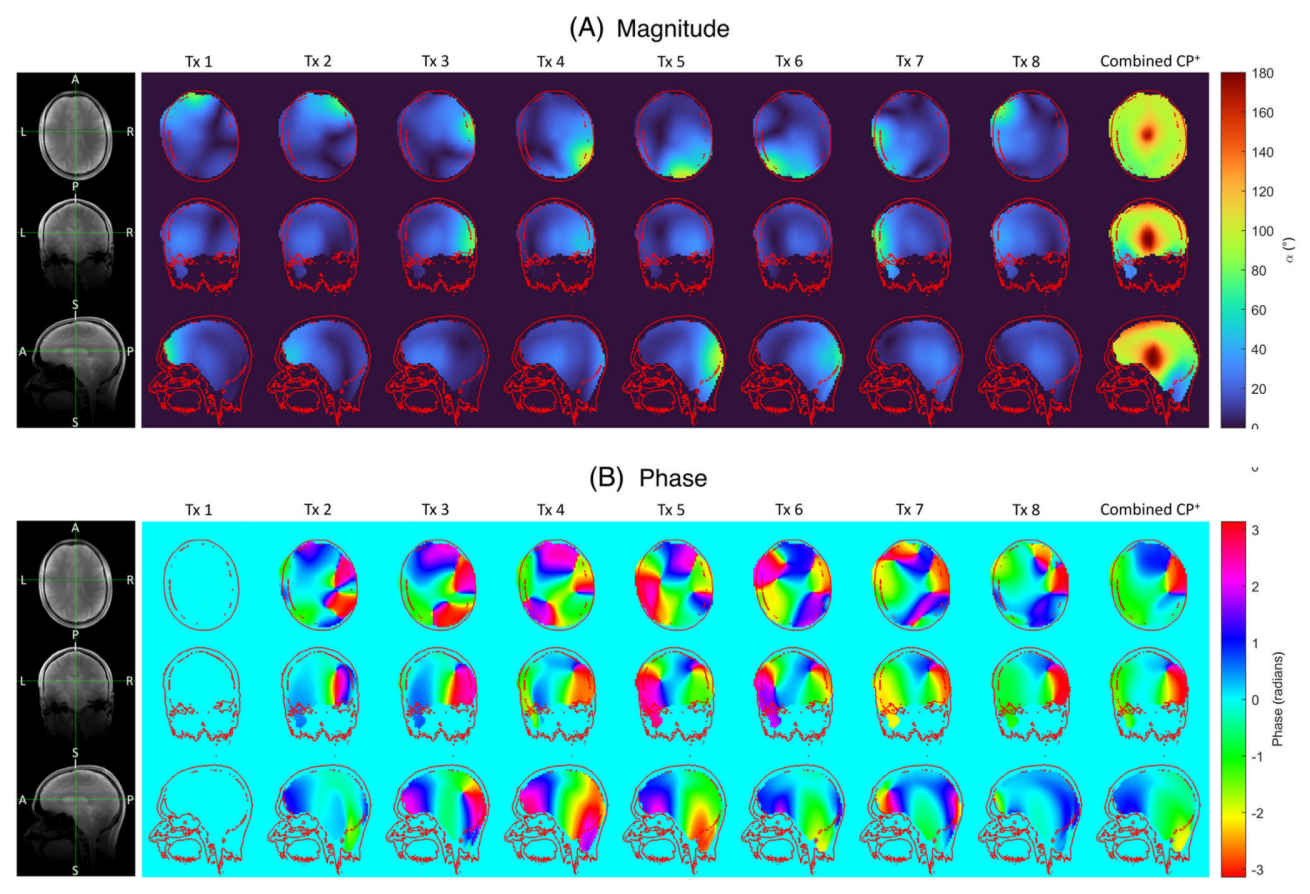
3D multi-channel and combined CP^+^ ${\text{B}}_{1}^{+}$ maps acquired in the brain of one subject are shown for axial, coronal, and sagittal planes for (A) magnitude and (B) phase. Data was acquired at a 60 V reference voltage. Maps are reconstructed to 64 × 64 × 64 from an acquired scan matrix of 24 × 24 × 24.

The magnitude and phase of the ${\text{B}}_{1}^{+}$ maps acquired in the brain are displayed in Figure 5(A,B), respectively. The mean flip angle in CP^+^ in the brain was 86° (min/-max = 19°/189°, CV = 30%). Additional subjects can be seen in Figure S8.

**Figure 6:**
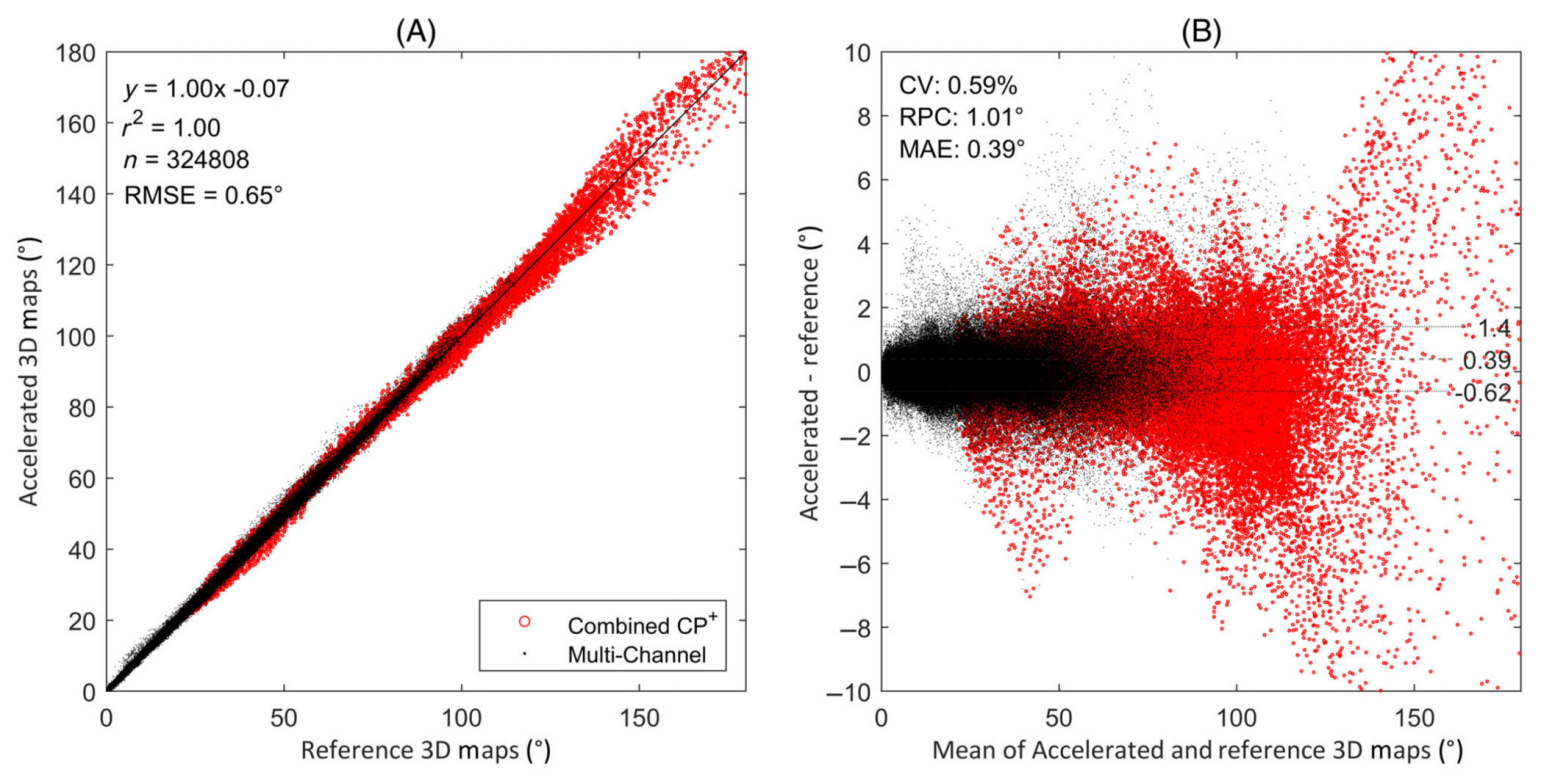
Correlation and Bland–Altman plots can be seen in (A) and (B), respectively, comparing the undersampled 3D dataset to a fully sampled reference across the entire masked volume in Figure 5. Individual channels (black markers) and combined CP^+^ (red markers) are plotted together. Statistics are shown for the individual (non-combined) maps. The dotted lines represent ±1.96 SD around the mean (solid line).

Figure 6(A,B) show a correlation and Bland–Altman plot comparing accelerated maps to the fully sampled reference. The maps show excellent linearity and good agreement with an RMSE of 0.7° across all multichannel maps to the reference and 1.9° when combined in CP^+^.

**Figure 7:**
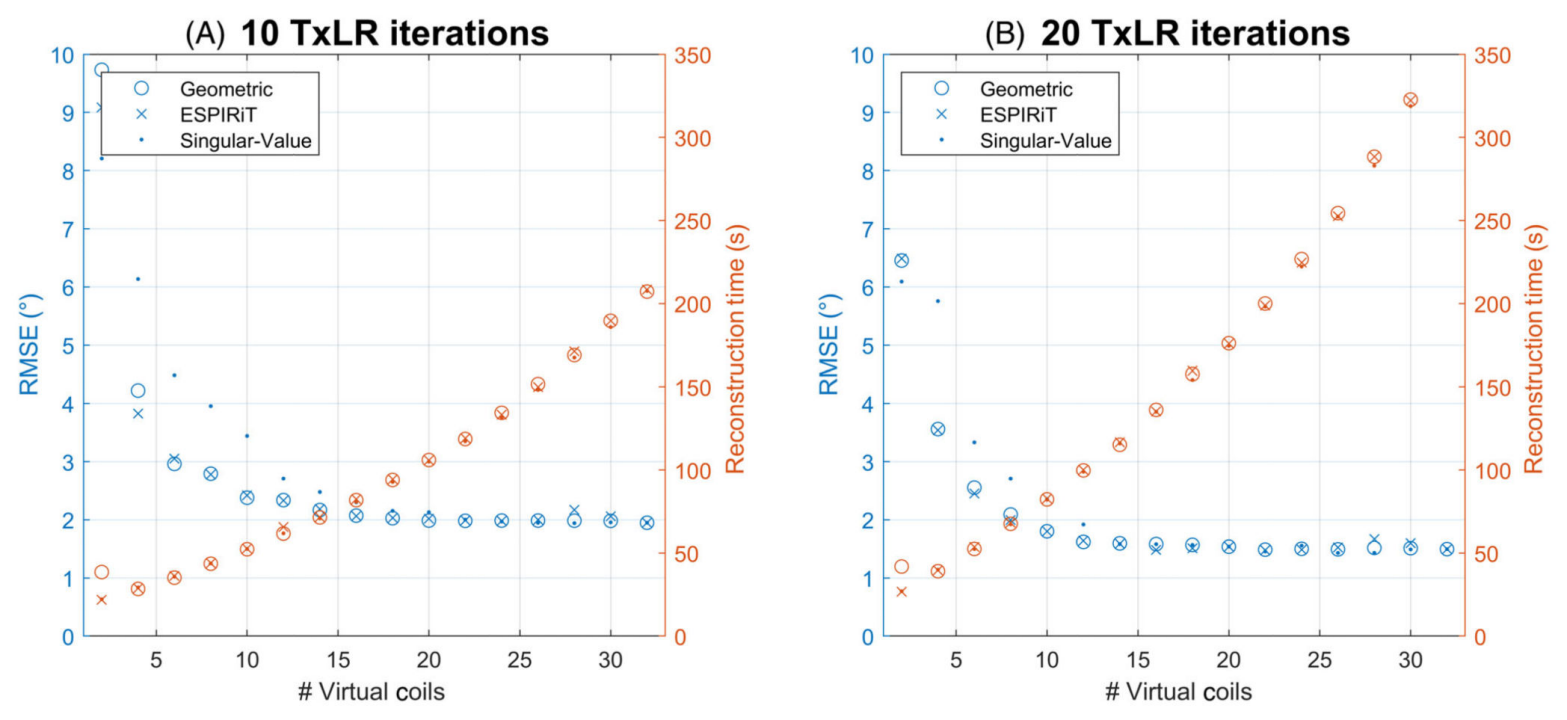
The effect of three coil-compression algorithms on the RMSE and reconstruction time for (A) 10 TxLR iterations and (B) 20 TxLR iterations for in vivo brain data acquired with an 8Tx/32Rx head coil. Tx/Rx, transmit/receive.

Coil-compression reduced the reconstruction time from over 6 min to 80 s using 10 virtual coils and 20 TxLR iterations or 50 s for 10 TxLR iterations, with only a small (0.6°) impact on RMSE to a fully sampled non-compressed reference, as seen in Figure 7

**Figure 8:**
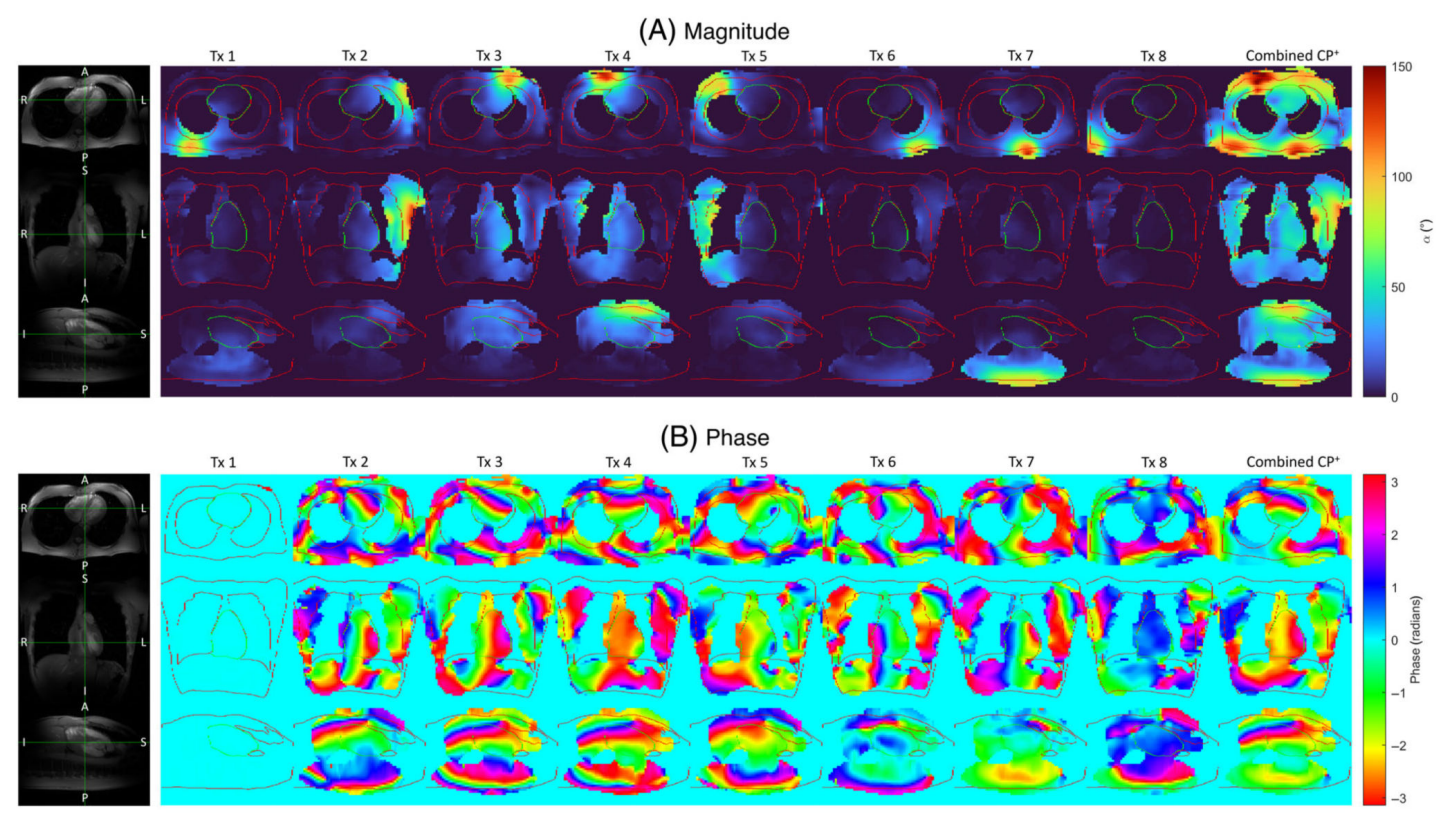
3D multi-channel and combined CP^+^ ${\text{B}}_{1}^{+}$ maps for (A) magnitude and (B) phase acquired in the body in 23 heartbeats shown for axial, coronal, and sagittal planes. Data was acquired at a 200 V reference voltage. Maps are reconstructed to 64 × 64 × 48 from an acquired scan matrix of 24 × 24 × 24. Body and heart boundaries are overlaid from the cine images.

Data acquired in the body of one subject is shown in Figure 8. The mean flip angle over the heart in CP^+^ is 42° (min/max = 6°/137°, CV = 29%). The poor cine image intensity in the right atrium is explained by the low ${\text{B}}_{1}^{+}$ magnitude of the combined CP^+^ map in this region. Additional subjects can be seen in Figure S9.

**Figure 9:**
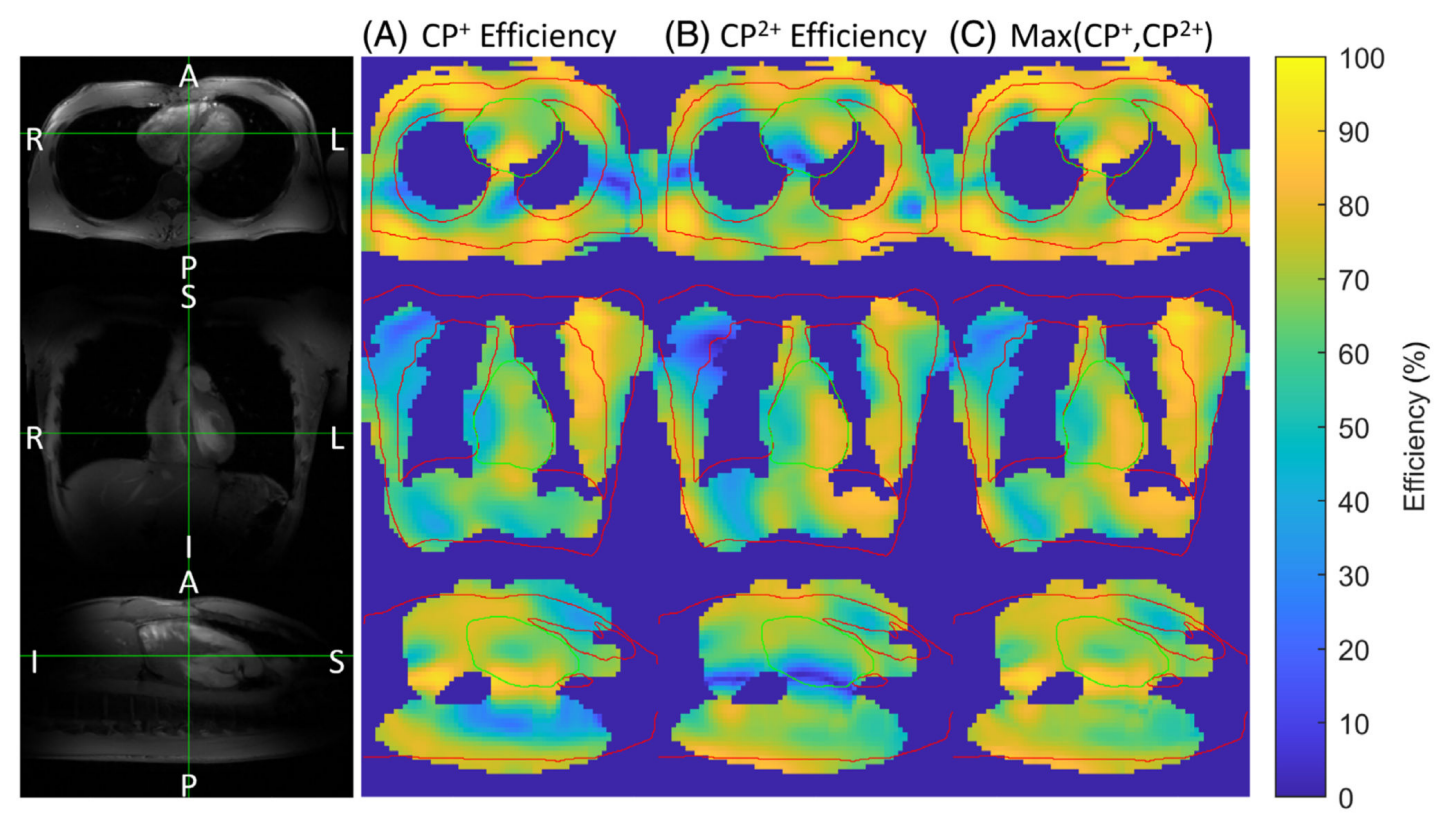
The shim efficiencies are shown for axial, coronal, and sagittal planes for (A) CP^+^ mode, (B) CP^2+^ mode, and (C) the maximum efficiency.

The shim efficiency for CP^+^, CP^2+^, and the maximum of both shim modes is shown in Figure 9. The combination of CP^+^ and CP^2+^ appears to be inadequate over the entire heart, particularly in the right atrium.

## 4 Discussion

We have demonstrated the feasibility of accelerating the acquisition of multi-channel absolute ${\text{B}}_{1}^{+}$ maps. We did this by combining three methods from the literature (TxLR, B1TIAMO, and Sandwich) and utilizing small 24 × 24 scan matrices to derive the dominant transmit fields for pTx calibration. The use of such matrices leverages the same underlying physical principles as for Rx-coil sensitivity calibration, which typically relies on comparably sized scan matrices. Simulations using high-resolution synthetic body data demonstrate that the largest source of error in the cropped maps is due to these small matrices not capturing the highest frequency fluctuations in the absolute ${\text{B}}_{1}^{+}$ from constructive/destructive interference. However, this error is small, with an RMSE of 3.8° in CP^+^ mode with a mean flip angle of 66° over the heart. Our simulation results using in silico heart data and retrospective undersampling of brain data show that we can comfortably obtain reliable undersampled ${\text{B}}_{1}^{+}$ maps in the head and body using acceleration factors of 4. We also found from simulations that using a small (4 × 4) calibration region was important for robust ${\text{B}}_{1}^{+}$ field map estimation at larger undersampling factors.

The results in Figure 5 demonstrate the ability to obtain good quality multi-channel ${\text{B}}_{1}^{+}$ maps in the brain in only 14 s, free of any structural artifacts except where there are signal voids such as in the sinuses. These maps are in agreement with the fully sampled reference, yielding an RMSE of 1.9° combined in CP^+^, and are consistent with retrospective undersampling results. Higher accelerations were achievable with the Nova (8Tx/32Rx) head coil over the dipole array (8Tx/8Rx) due to additional Rx channels present. However, higher accelerations for the relative mapping portion of the sequence were not worth-while due to only a minor additional scan time of 1 s for *R* = 4 versus *R* = 6.

Figure 8 shows the multi-channel ${\text{B}}_{1}^{+}$ maps through axial, coronal, and sagittal slices of the body obtained in only 23 heartbeats. A fully sampled acquisition is unfeasible because it would require around 86 heartbeats and several breath-holds. The current implementation required the acquisition in two separate breath-holds due to sequence preparation time. We are currently working on an implementation for our updated VE12U system, which will acquire both relative and absolute maps within a single sequence, enabling single breath-hold acquisitions. To our knowledge, this method enables multi-channel ${\text{B}}_{1}^{+}$ mapping in the heart within a single breath-hold for the first time. Further pulse sequence optimization may need to be performed because 23 heartbeats could still be too long for patients suffering from breathlessness. To overcome this, maps could be acquired in two separate breath-holds of around 17 heartbeats, where additional time could be spent mapping additional modes. The ${\text{B}}_{1}^{+}$ maps shown here were acquired in the heart of subjects with small chests, and they have not been directly validated due to the lack of a reliable ground-truth in the body. The best gold standard we have available is in silico body data and the simulations we performed, along with retrospective results in the brain and prospective results in phantoms and the brain, which provide us with confidence in the validity of these maps.

We expect our maps to be more robust to motion due to the short acquisition time during diastole. However, there are some artifacts present in the magnitude of the multi-channel maps, which are present where the maximum shim efficiency of CP^+^ and CP^2+^ is low. Therefore, although CP^+^ and CP^2+^ appear to work well in the brain, this work suggests that CP^+^ and CP^2+^ shim modes, with the dynamic range of the Sandwich method, are not optimal for 3D ${\text{B}}_{1}^{+}$ mapping in the body; an additional shim mode or additional voltages^27^ may be required in the body, which warrants further investigation. Alternatively, a universal shim set^31^ could be used, or as discussed in the original B1TIAMO paper, a dedicated heart shim could be calculated from the relative maps, but this would require additional processing time. Higher acceleration factors could be explored for cardiac coils with more than eight Rx channels.

This method is conceptually simple to apply to larger transmit arrays. In the brain, a 16-channel transmit system would require a scan time of approximately 18 s and a 32-channel transmit system around 26 s, without considering further acceleration. For imaging the heart with larger transmit arrays, more heartbeats will be needed in the relative mapping portion of the sequence and may require multiple breath-holds. The main limitation of this acquisition scheme is currently the long reconstruction time, with the average offline reconstruction times for the chest/head coils being 1/6 min using our current hardware. However, this has not been optimized, nor is it prohibitive, and there are several potential avenues to reduce this. One such possibility is the use of coil compression.^32^ Initial tests with a geometric-decomposition coil compression algorithm^33^ were able to reduce brain reconstruction times from 6 min to around 1 min (Figure 7). Alternatively, a more efficient method of computing the singular value decomposition, which accounts for a significant portion of the computation time in the TxLR algorithm or a machine learning approach, could be investigated.

## 5 Conclusion

We have demonstrated a method to accelerate the acquisition of 3D multi-channel ${\text{B}}_{1}^{+}$ maps that is fast and has good agreement with a fully sampled reference in the brain. This method was applied to obtain 3D multi-channel maps in the body and is important to help facilitate cardiac imaging at 7 T.

## Supplementary Material

**Supporting Information**

Additional supporting information may be found in the online version of the article at the publisher’s website.

Supplementary

## Figures and Tables

**Table 1:** Sequence parameters used in vivo and in phantom to acquire the accelerated multi-channel ${\text{B}}_{1}^{+}$ maps.

Target	Coil	FOV (mm^3^)	Scan matrix	Relative Acq.				Absolute Acq.						Ref Voltage (V)	BW (Hz/px)	Acq. Time
				*R*	TE_GRE_ (ms)	TR_GRE_ (ms)	λ (°) (RECT)	R	TE_FLASH_ (ms)	TR_FLASH_ (ms)	TR_LONG_	*α* (°) (HS8)	*β* (°) (RECT)			
“Braino” Phantom	8Tx/32Rx	250 × 250 × 250	24^3^	4	1.31	3.14	9	6	1.26	3	1 s	90	5	60	490	14 s
Drum Phantom	8Tx/8Rx	300 × 300 × 300	24^3^	4	1.31	3.14	9	4	1.26	3	700 ms	90	5	200	490	16 s or 23 HB
Brain	8Tx/32Rx	250 × 250 × 250	24^3^	4	1.31	3.14	9	6	1.26	3	1 s	90	5	60	490	14 s
Heart	8Tx/8Rx	380 × 380 × 285	24^3^	4	1.31	3.14	9	4	1.26	3	1 HB	90	5	200	490	23 HB

*Note*: BW, bandwidth; GRE, gradient echo; HB, heartbeat; HS, hyperbolic secant; Tx/Rx, transmit/receive.

## Data Availability

MATLAB code (GitHub hash: e49c1a8) for generating and plotting the simulation results (Figures 2 and 3, and Supporting Figures S3 and S4) using the synthetic body images is available at https://github.com/jameslewiskent/Acc3DmTxMapping.
