# Supplementary material for: Accelerated 3D multi-channel B1+ mapping at 7 T for the brain and heart
Source: Magn Reson Med. Author manuscript; Available in PMC 2026 Mar 14. (PMC7618857; doi:10.1002/mrm.30201)
Supplement: Supplementary [file EMS206316-supplement-Supplementary.docx]

**Supporting Information for**

‘Accelerated 3D Multi-Channel B_1_^+^ Mapping at 7T for the Brain and Heart’

James L. Kent^a^, Matthijs H.S. de Buck^b,c,d^, Iulius Dragonu^e^, Mark Chiew^a,f,g^, Ladislav Valkovič^h,i^, and Aaron T. Hess^a^

^a^ Wellcome Centre for Integrative Neuroimaging, FMRIB, Nuffield Department of Clinical Neurosciences, University of Oxford, Oxford, United Kingdom
^b^ Spinoza Centre for Neuroimaging, Amsterdam, Netherlands
^c^ Computational Cognitive Neuroscience and Neuroimaging, Netherlands Institute for Neuroscience, KNAW, Amsterdam, Netherlands
^d^ Department of Radiology and Nuclear Medicine, Amsterdam University Medical Centers, University of Amsterdam, Amsterdam, Netherlands.
^e^ Research & Collaborations GB&I, Siemens Healthcare Ltd, Camberley, United Kingdom
^f^ Department of Medical Biophysics, University of Toronto, Toronto, Canada
^g^ Physical Sciences, Sunnybrook Research Institute, Toronto, Canada
^h^ Oxford Centre for Clinical Magnetic Resonance Research (OCMR), University of Oxford, Oxford, United Kingdom
^i^ Department of Imaging Methods, Institute of Measurement Science, Slovak Academy of Sciences, Bratislava, Slovakia


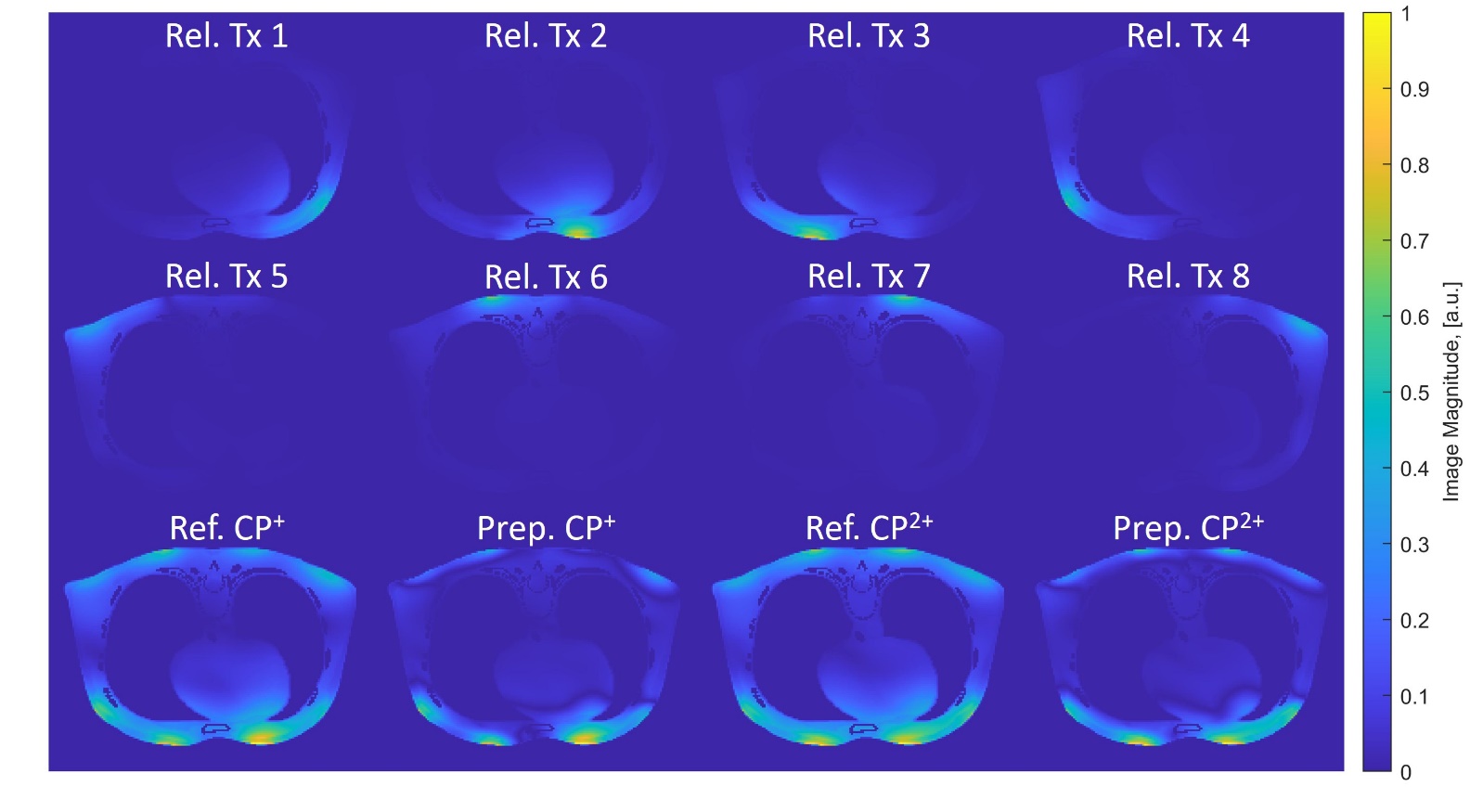
Figure S1: The fully sampled native resolution (139×178) synthetic images (coil-combined) used to test various acceleration factors for an 8-transmit 8-receive dipole array chest coil at 7T.

**
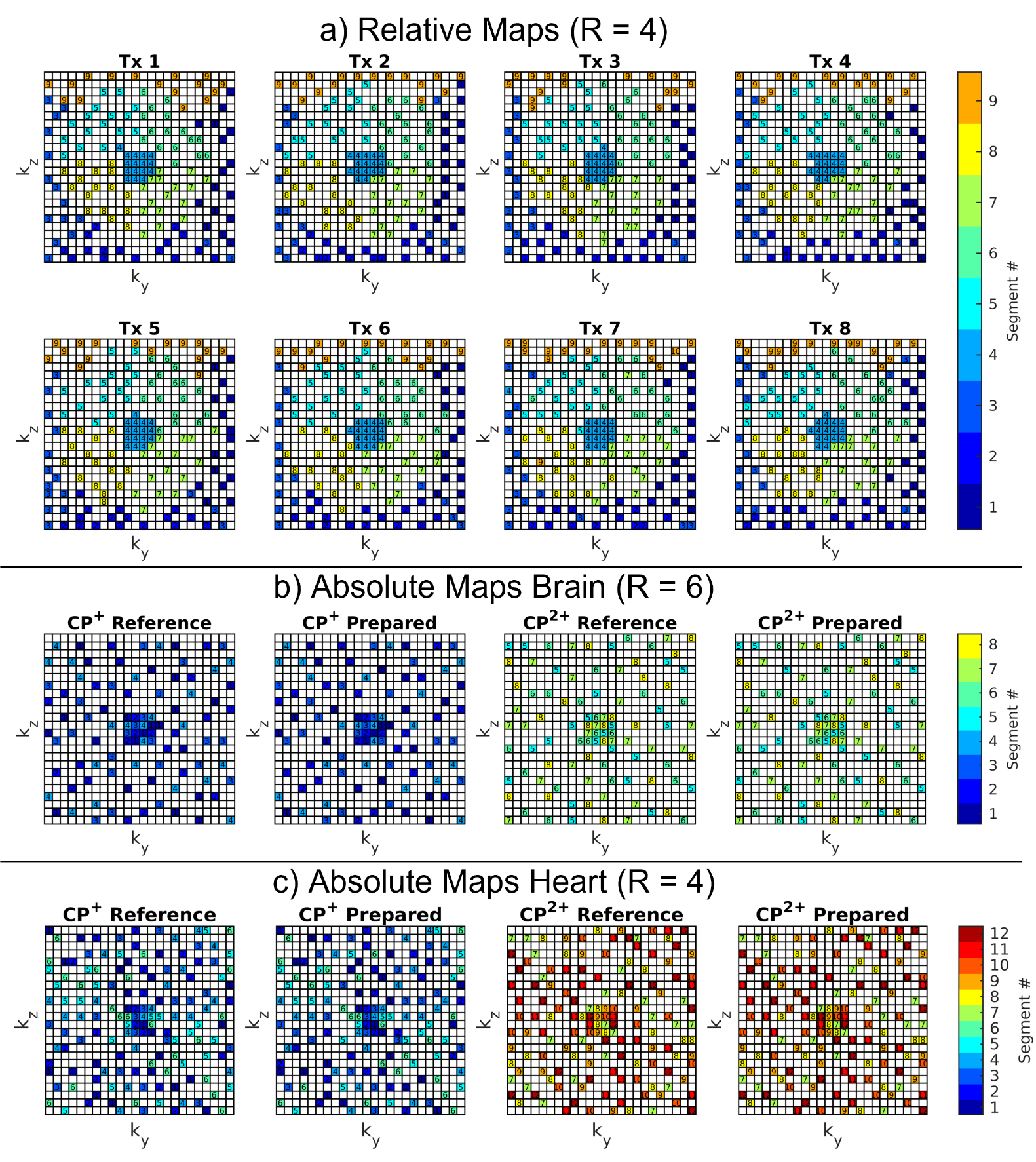
**Figure S2: The undersampling masks used for **(a)** relative maps with an acceleration factor R = 4 **(b)** absolute maps in the brain with an acceleration factor R = 6 and **(c)** absolute maps in the heart with an acceleration factor R = 4. The colours indicate the segment during which the readout line was acquired.


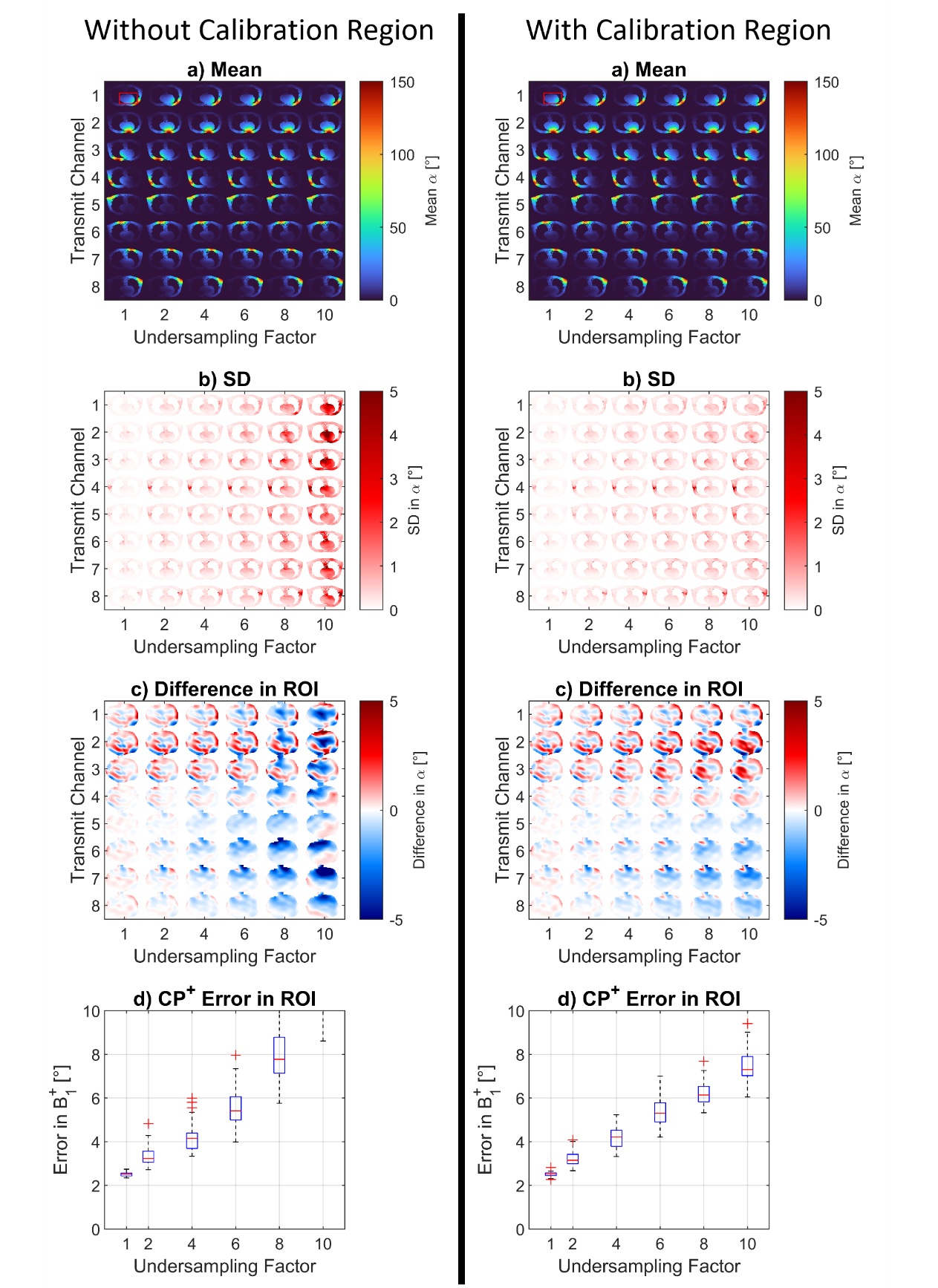

Figure S3: The effect of not acquiring a calibration region is shown (left) in comparison to acquiring a small 4x4 calibration region (right). Mean **(a)** and SD **(b)** for multi-channel parallel transmit B_1_^+^ maps across various undersampling factors (1-16). 50 repeats with different undersampling masks were performed. Subfigure **(c)** shows the mean difference across these repeats to the fully sampled k-space in a region of interest, the results of which are summarised by the RMSE **(d)**. The small red box in the top left of subfigure **(a)** indicates the heart ROI used for plots **(c)** and **(d)**.


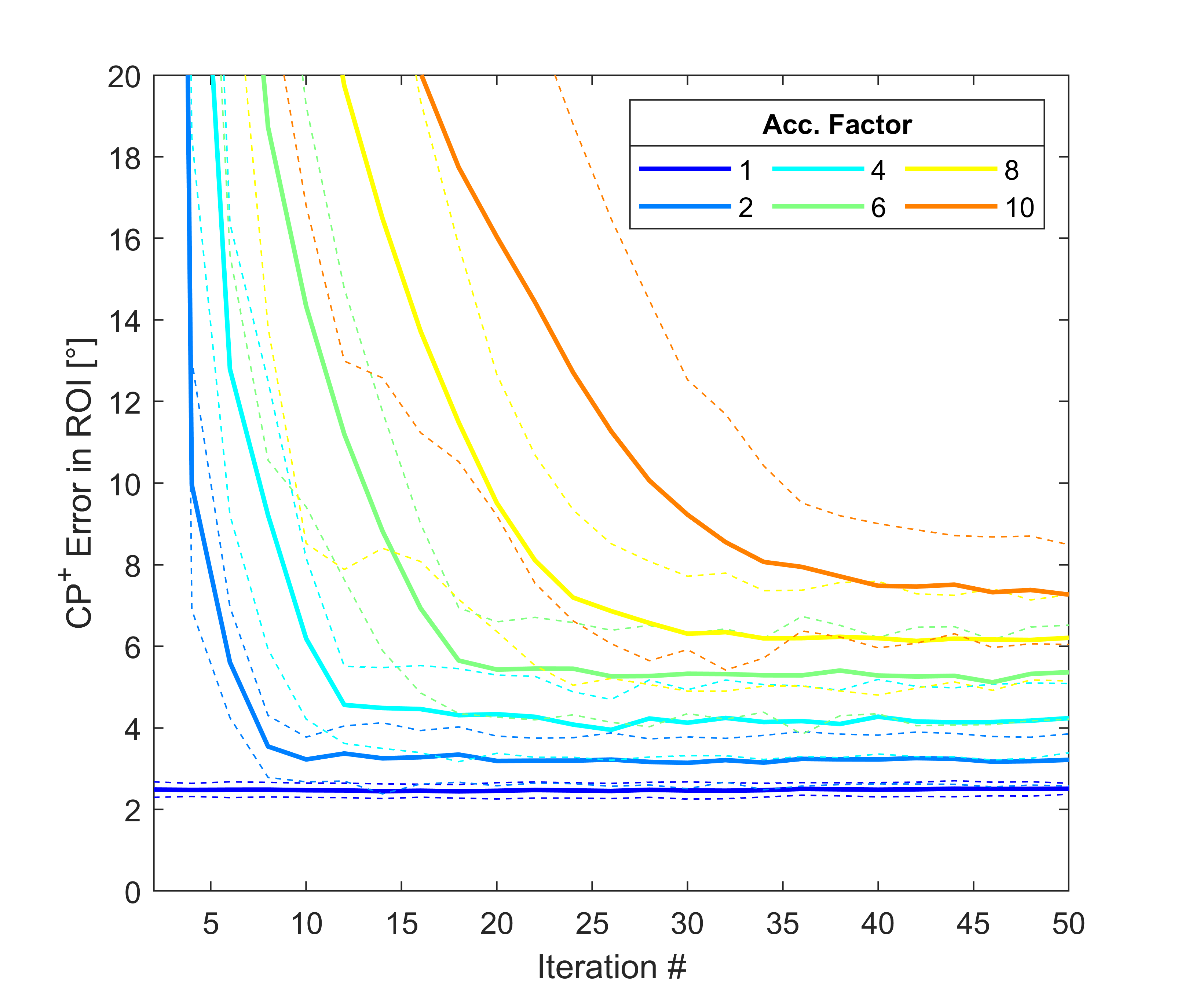

Figure S4: The number of TxLR iterations versus the root-mean-square error in the combined CP^+^ B_1_^+^ maps to the fully sampled native resolution (139×178) ground truth based on data simulated using synthetic body images. Dashed lines indicate ±1.96SD.


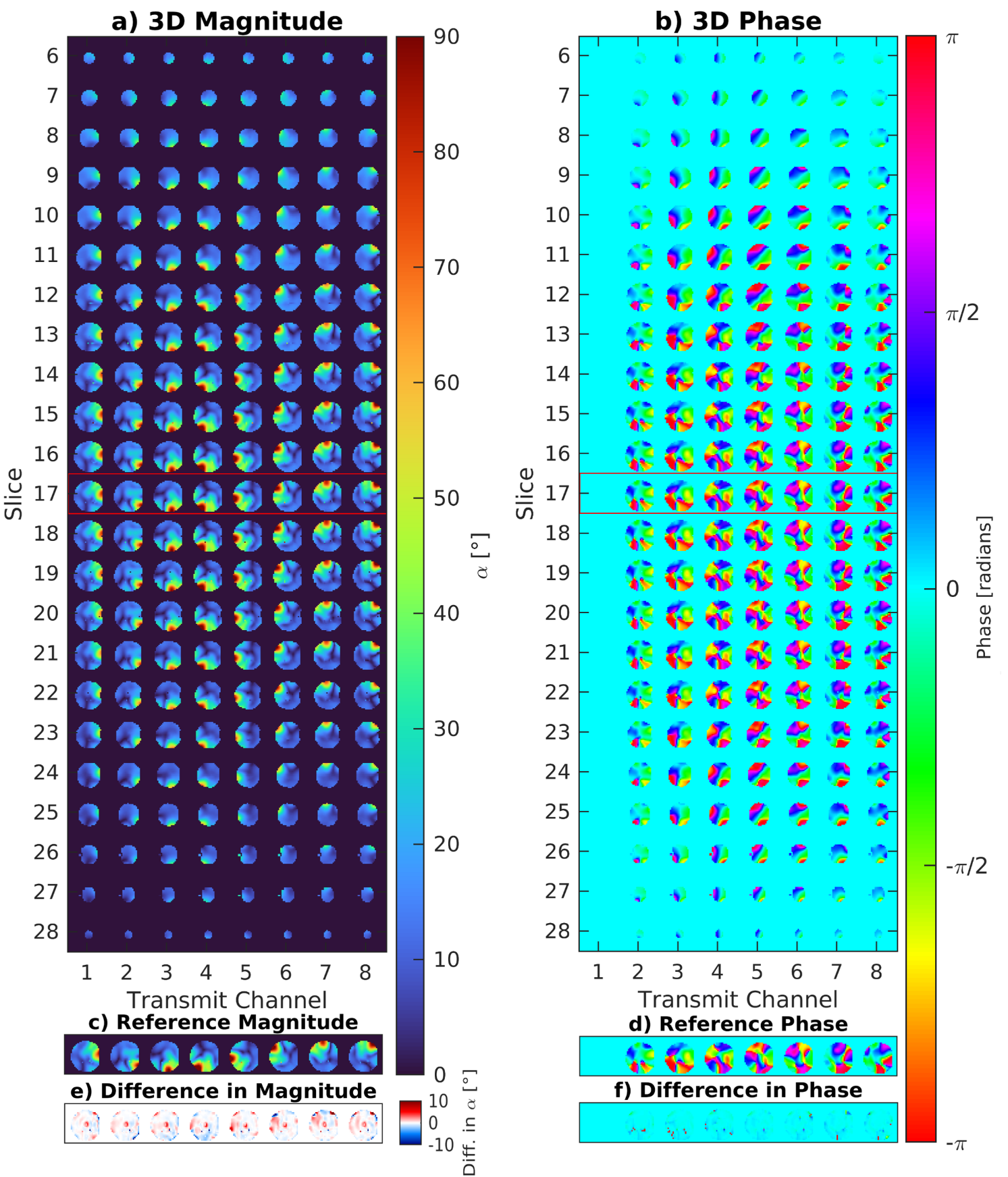

Figure S5: 3D multi-channel maps in a spherical water phantom acquired in 14 s using an 8Tx/32Rx head coil. The proposed method is shown for both magnitude **(a)** and phase relative to transmit channel 1 **(b)**. A slice from the fully sampled reference multi-channel maps is also shown for both magnitude **(c)** and phase **(d)**. The difference between a central slice (outlined in red in **(a)**) is shown for both magnitude **(e)** and phase **(f)**.


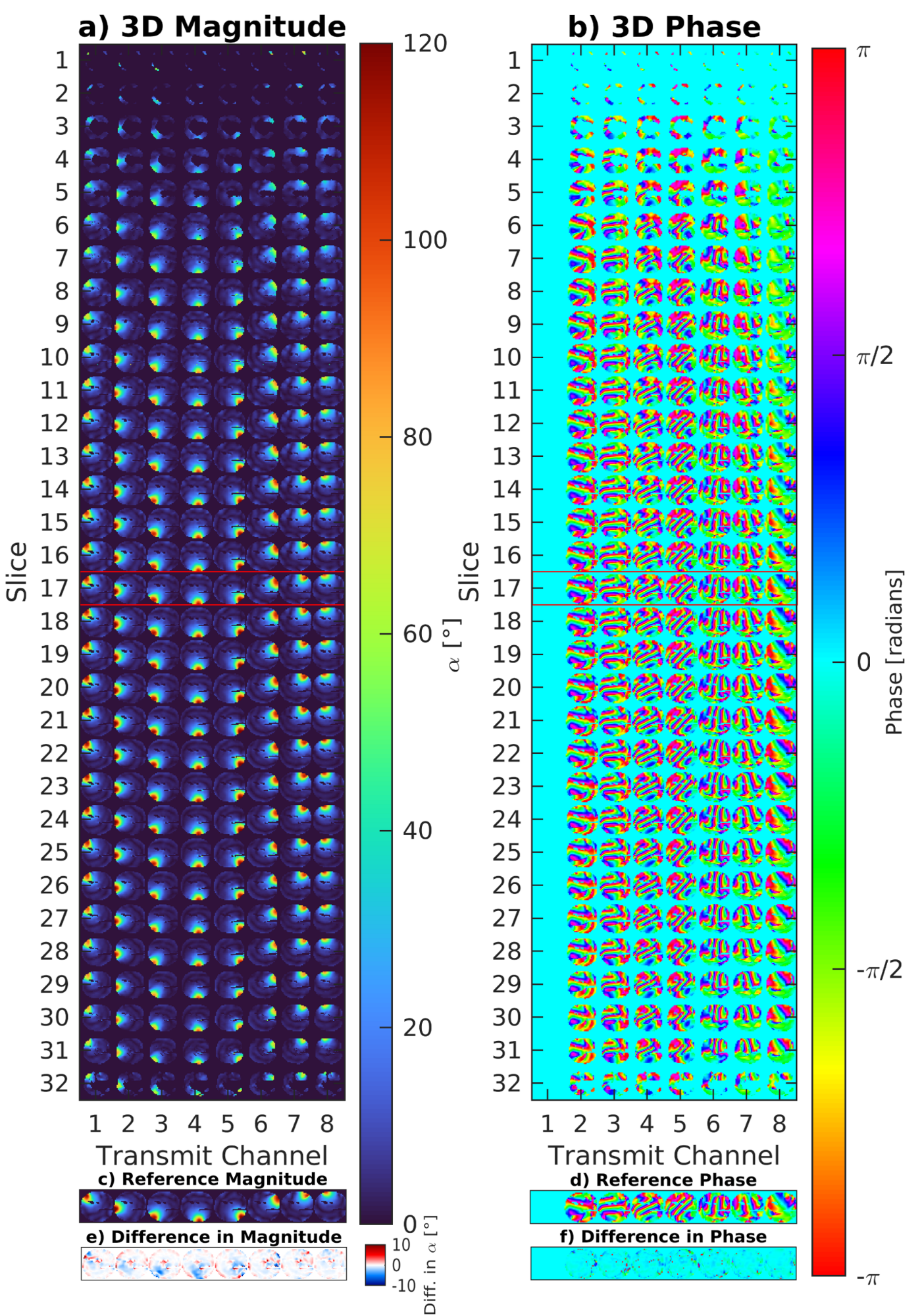

Figure S6: 3D multi-channel maps in a large drum water phantom acquired gated in 23 simulated heartbeats (RR interval = 700 ms) using an 8Tx/8Rx dipole-array chest coil. The proposed method is shown for both magnitude a) and phase relative to transmit channel 1 **(b)**. A slice from the fully sampled reference multi-channel maps is also shown for both magnitude **(c)** and phase **(d)**. The difference between a central slice (outlined in red in **(a)**) is shown for both magnitude **(e)** and phase **(f)**.


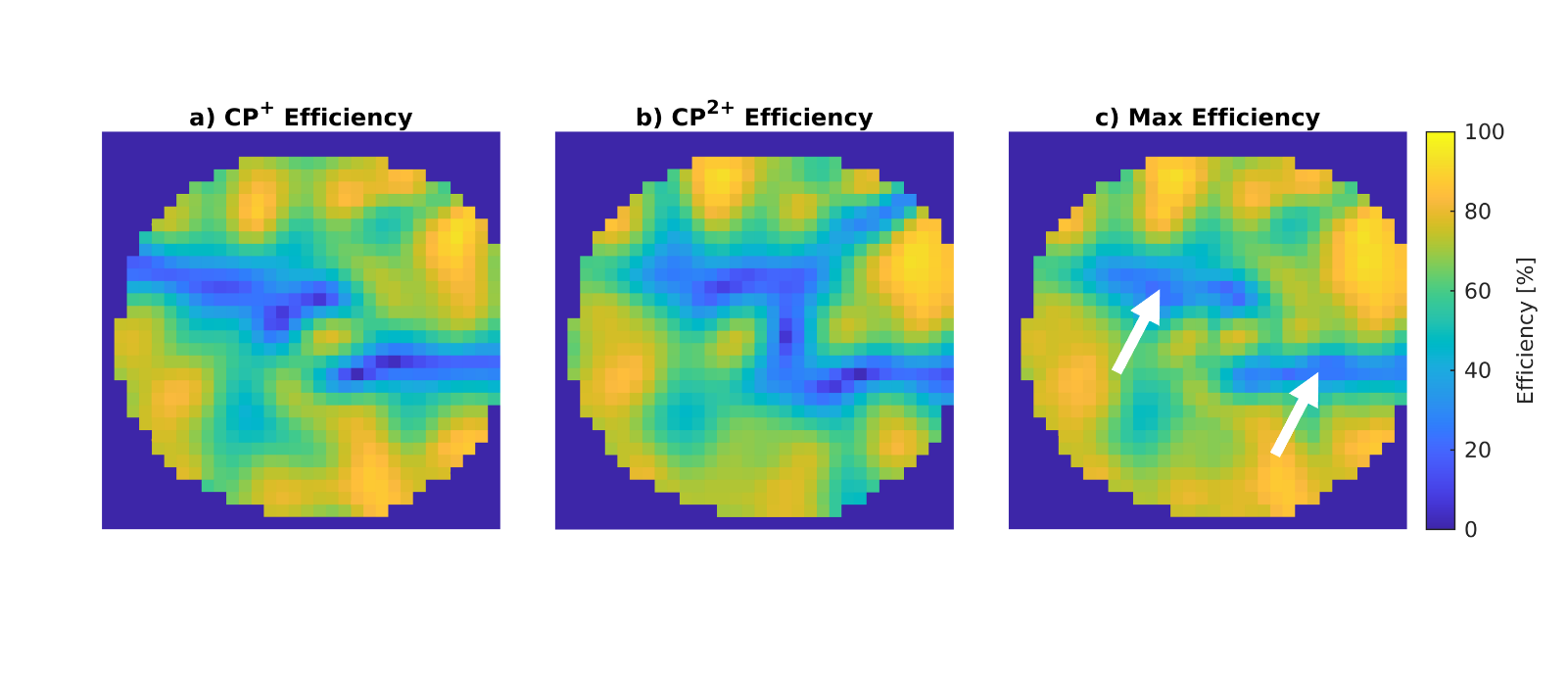


Figure S7: The shim efficiencies are shown for a slice from the drum phantom for a) CP^+^ mode, b) CP^2+^ mode and c) the maximum efficiency used for additional masking. As indicated by the white arrows, CP^+^ and CP^2+^ do not appear to provide sufficient coverage in this phantom.

**
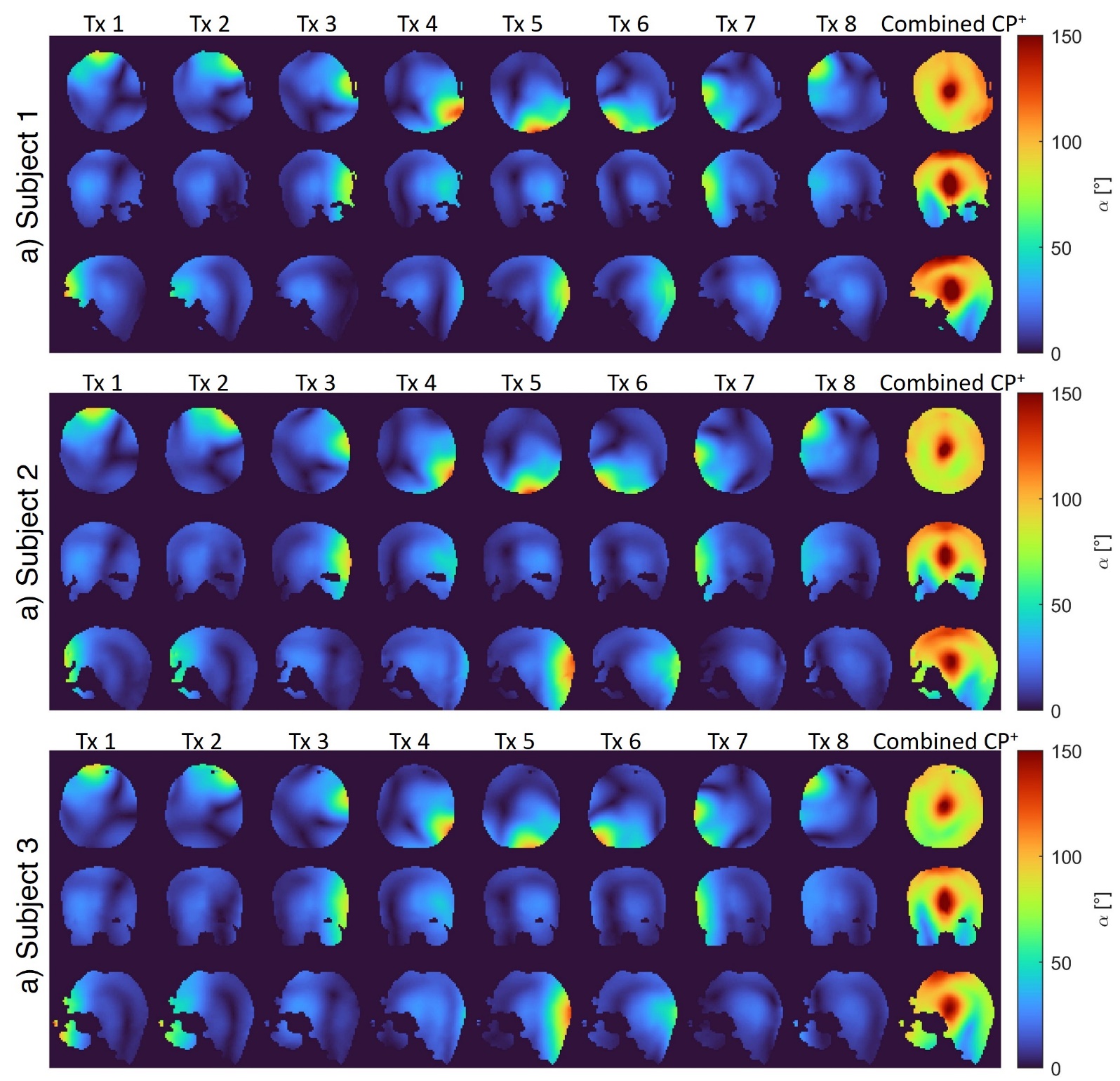
**Figure S8: The magnitude of 3D multi-channel and combined CP^+^ B_1_^+^ maps for three subjects acquired in the brain shown for axial, coronal and sagittal planes. Data was acquired at a 60 V reference voltage. Maps are reconstructed to 64×64×64 from an acquired scan matrix of 24×24×24.


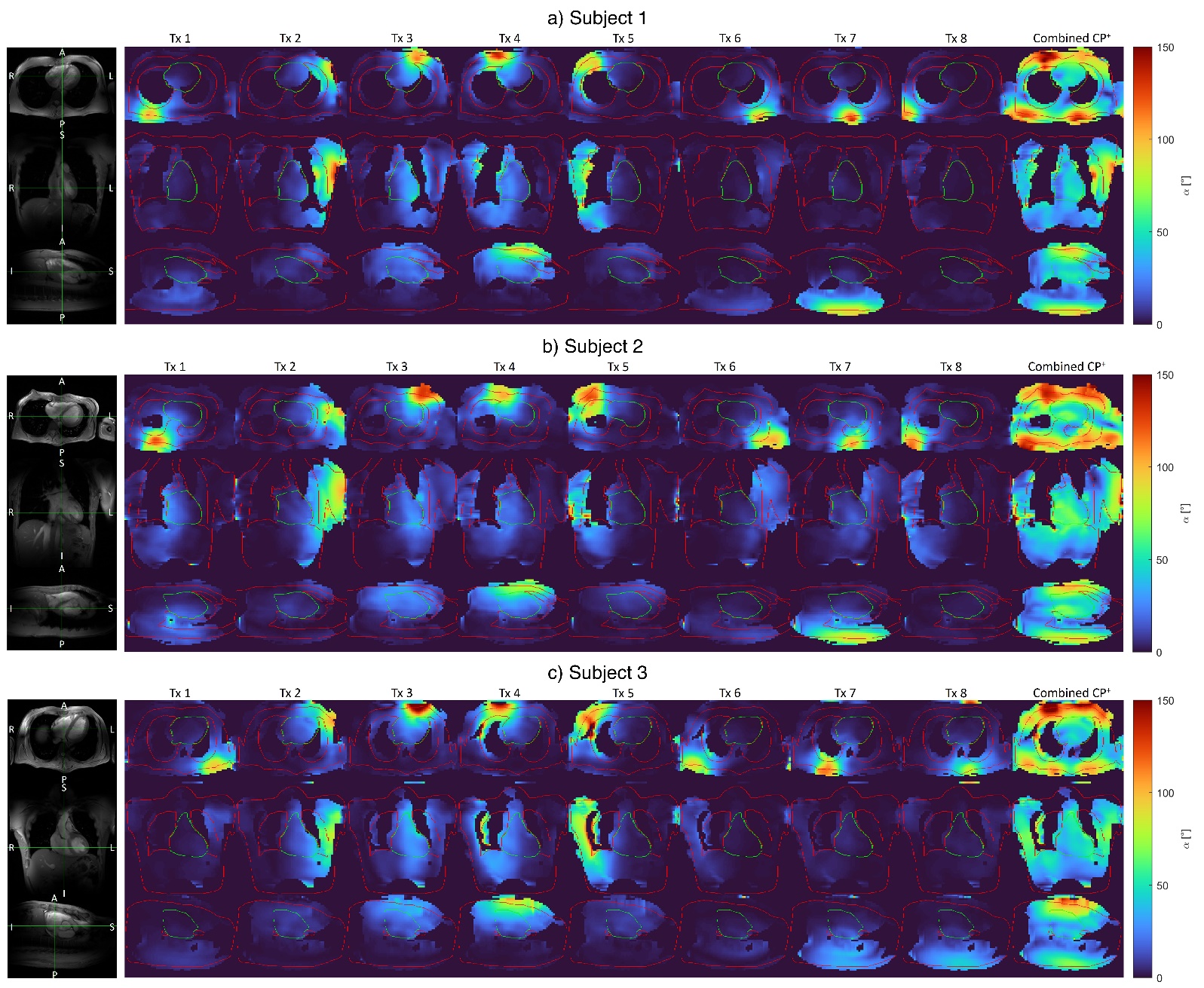
Figure S9: The magnitude of 3D multi-channel and combined CP^+^ B_1_^+^ maps for three subjects acquired in the body in 23 heartbeats shown for axial, coronal and sagittal planes. Data was acquired at a 200 V reference voltage. Maps are reconstructed to 64×64×48 from an acquired scan matrix of 24×24×24. Body and heart boundaries are overlayed from the cine images. Note: The placement of the posterior elements for subject 3 were unintentionally reversed.
